# Long noncoding RNA FTX ameliorates hydrogen peroxide-induced cardiomyocyte injury by regulating the miR-150/KLF13 axis

**DOI:** 10.1515/biol-2020-0100

**Published:** 2020-12-31

**Authors:** Yamin Zhang, Xiaoying Fan, Hua Yang

**Affiliations:** Department of Cardiology, The First Affiliated Hospital of Airforce Military Medical University, No. 127, Changle West Road, Xi’an, Shaanxi, 710032, China

**Keywords:** I/R injury, lnc FTX, miR-150, KLF13, progression

## Abstract

**Background:**

Myocardial reperfusion is an effective therapy for acute myocardial infarction (AMI). However, ischemia/reperfusion (I/R) injury following myocardial reperfusion is a significant limitation for AMI treatment. Five prime to Xist (FTX) was recognized as a biomarker of multiple diseases, including heart disease. However, the molecular mechanism of FTX in I/R injury is unclear.

**Methods:**

Cell viability was evaluated by using cell counting kit-8 (CCK-8) assay. Apoptosis was analyzed by using a caspase-3 activity detection kit and flow cytometry. The expression of FTX, microRNA (miR)-150, and Kruppel-like factor 13 (KLF13) was measured by quantitative reverse transcription-polymerase chain reaction (qRT-PCR). The interaction of miR-150 and FTX or KLF13 was confirmed by a dual-luciferase reporter and RNA immunoprecipitation (RIP) assays. Protein expression of KLF13 was examined by Western blot. The role of FTX was detected in I/R-injured heart tissues *in vivo*.

**Results:**

Hydrogen peroxide (H_2_O_2_) induced cardiomyocyte injury by decreasing cell viability and expediting cell apoptosis. However, FTX alleviated cardiomyocyte injury by promoting cell proliferation and restricting cell apoptosis of H9C2 cells that were treated with H_2_O_2_. In addition, we discovered that FTX directly interacted with miR-150, while KLF13 was a target of miR-150. Rescue experiments showed that miR-150 neutralized the FTX-mediated promotion of cell progression and restriction of cell apoptosis in H9C2 cells treated with H_2_O_2_. KLF13 knockdown restored the effect of miR-150 on increased proliferation and decrease in apoptosis in H_2_O_2_-treated cardiomyocytes. Furthermore, FTX enhanced the expression of KLF13 protein through interaction with miR-150. Upregulation of FTX repressed apoptosis in I/R-injured heart tissues *in vivo*.

**Conclusion:**

FTX relieves H_2_O_2_-induced cardiomyocyte injury by increasing KLF13 expression via depletion of miR-150, thus providing a novel therapeutic target for the alleviation of I/R injury.

## 1 Introduction

Acute myocardial infarction (AMI) is a serious cardiovascular disease accompanied with quadriplegia or paraplegia [1]. AMI pathogenesis is complicated and includes factors such as obesity, sedentary lifestyle, diabetes, smoking, and dyslipidemia. Myocardial reperfusion has dramatically improved the therapeutic outcomes of AMI patients [2,3]. Unfortunately, myocardial reperfusion can often lead to ischemia/reperfusion (I/R) injury and initiate oxidative stress, ventricular arrhythmias, cardiomyocyte death, and neuronal apoptosis [4,5,6]. Therefore, it is imperative to clarify the molecular mechanism of I/R injury-induced cardiomyocyte injury.

Long noncoding RNAs (lncRNAs) are critical modulators of many diseases through their impact on gene expression [7]. LncRNA five prime to Xist (FTX), located at X-chromosome inactivation center, is associated with the pathogenesis of multiple diseases, such as epilepsy, heart disease, and cancers [8,9]. For example, FTX accelerated cirrhotic patients’ inflammatory response by activating macrophages through the depletion of miR-545 and regulation of Tim-3 expression [10]. In addition, dysregulation of FTX has been observed in a variety of cancers. FTX acts as an oncogene to increase cell growth, colony formation, and invasion by enhancing AEG-1 expression via targeting miR-342-3p in glioma [11]. By contrast, the elimination of FTX reduced cell cycling, proliferation, and colony formation in renal cell carcinoma [12]. However, the function of FTX in cardiomyocyte injury is still poorly understood.

MicroRNAs (miRNAs) are conserved transcripts that participate in many pathological processes, such as cell cycle, metabolism, growth, differentiation, migration, inflammation, and apoptosis [13,14,15]. Ectopic expression of miRNAs was identified as a major cause of different diseases [16]. For example, miR-150 is a marker of sepsis and has been shown to reduce inflammation and apoptosis of umbilical vein endothelial cells by regulating NF-κB1 [17]. The abundance of miR-150 could target AKT3 and alleviate LPS-induced lung injury by regulating cell viability, inflammation, autophagy, and apoptosis through the JNK/NF-κB pathway [18]. In addition, miR-150 behaved as a tumor suppressor to inhibit cell growth in non-small cell lung cancer or melanoma by interacting with EPG5 or MYB [19,20]. These findings prompted us to consider that miR-150 may play an important role during cardiomyocyte injury.

Kruppel-like factor 13 (KLF13) is a member of the KLF families, which is overexpressed in the heart [21]. Inhibition of KLF13 has been reported to protect the heart from AMI through its interaction with miR-125b-5p [22]. Therefore, KLF13 may also be involved in cardiomyocyte protection after I/R injury.

We attempted to illuminate the regulatory mechanism of FTX in the cardiomyocyte response to I/R injury. Hydrogen peroxide (H_2_O_2_) treatment of cardiomyocytes was utilized to mimic I/R injury. The influences of FTX, miR-150, and KLF13 on H_2_O_2_-treated cardiomyocytes were evaluated by rescue experiments.

## 2 Materials and methods

### 2.1 Cell culture and treatment

Cardiac-derived H9C2 cells isolated from rats were purchased from American Type Culture Collection (ATCC, Manassas, VA, USA) and cultured in complete Dulbecco’s modified Eagle medium (Gibco, Carlsbad, CA, USA) supplemented with 10% fetal bovine serum (cat. no. 10099; Gibco). Cells were cultured in a constant temperature biochemical incubator (Thermo Scientific, Waltham, MA, USA) at 37°C in 5% CO_2_. The H9C2 cells were treated with H_2_O_2_ (0, 50, 100, and 200 µM) for 24 h. H9C2 cells treated with 100 µM H_2_O_2_ were transfected with different vectors for 48 h.

### 2.2 Cell counting kit-8 (CCK-8) assay

H9C2 cells were plated onto 96-well plates overnight. After H_2_O_2_ treatment for 24 h and cell transfection for 48 h, 10 µL of the CCK-8 reagent (Beyotime, Shanghai, China) was added to the H9C2 cells and left for 2 h. Finally, an optical density (OD) value of 490 nm was detected by a spectrophotometer (Thermo Scientific).

### 2.3 Caspase-3 activity detection

H9C2 cells were plated onto 24-well plates overnight, treated with H_2_O_2_ for 24 h and transfected with vectors for 48 h. After washing three times with phosphate buffer saline, the H9C2 cells were collected and stained with a caspase-3 activity detection kit (Beyotime). Finally, the caspase-3 activity of the H9C2 cells was measured by a spectrophotometer (Thermo Scientific).

### 2.4 Flow cytometry

H9C2 cells grown in 24-well plates were treated with H_2_O_2_ and transfected with different vectors. Then the H9C2 cells were collected and stained with fluorescein isothiocyanate-tagged annexin V/propidium iodide detection kit (Invitrogen, Carlsbad, CA, USA). The apoptotic rate was analyzed by a flow cytometer (ACEA NovoCyte; Agilent BIO, Santa Clara, CA, USA).

### 2.5 Quantitative reverse transcription-polymerase chain reaction (qRT-PCR)

RNA extraction from H9C2 cells was performed using TRIzol reagent (Invitrogen) following the manufacturer’s instructions. Next the cDNA for FTX and KLF13 was synthesized by All-in-One First-Strand cDNA Synthesis kit (GeneCopoeia, Guangzhou, China), while miR-150 was reverse transcribed with an All-in-One™ miRNA First-Strand cDNA Synthesis kit (GeneCopoeia). qPCR was performed using a Fast SYBR™ Green Master Mix (Applied Biosystems, Foster City, CA, USA) with a CFX Touch real-time PCR instrument (Bio-Rad, Hercules, CA, USA). Relative expression was calculated using the 2^−ΔΔCt^ method. Glyceraldehyde-3-phosphate dehydrogenase (GAPDH) and U6 were employed as internal references. The primers for FTX, miR-150, KLF13, GAPDH, and U6 are listed as follows: FTX (forward, 5′-TATGCCACCTAGCCTTTCTACA-3′; reverse, 5′-ATCTCTTCAAAAGCGGCATAAT-3′); miR-150 (forward, 5′-GCGTCTCCCAACCCTTGTA-3′; reverse, 5′-GTGCAGGGTCCGAGGT-3′); KLF13 (forward, 5′-CCGCAGAGGAAGCACAA-3′; reverse, 5′-CTTCTTCTCGCCCGTGT-3′); GAPDH (forward, 5′-AGGTCGGTGTGAACGGATTTG-3′; reverse, 5′-GGGGTCGTTGATGGCAACA-3′); and U6 (forward, 5′-ACCCTGAGAAATACCCTCACAT-3′; reverse, 5′-GACGACTGAGCCCCTGATG-3′).

### 2.6 Cell transfection

Small interfering RNA (siRNA) targeting FTX (si-FTX: 5′-GCTAGAACATCCCGAACTA-3′), siRNA targeting KLF13 (si-KLF13: 5′-GGCAGGACTGCAACAAGAA-3′), control (si-con: 5′-TGCACTGTGCAAGCCTCTTAA-3′), pcDNA, and FTX overexpression vectors (the sequences of FTX were inserted into pcDNA vector termed FTX) were synthesized by Genepharma (Shanghai, China). MiR-150 mimics (miR-150), miR-150 inhibitor (anti-miR-150), control (miR-con), and control inhibitor (anti-miR-con) were obtained from RIBOBIO (Guangzhou, China). The vectors were transfected into H9C2 cells by Lipofectamine 2000 (Invitrogen, Thermo Scientific).

### 2.7 Dual-luciferase reporter assay

The interaction between miR-150 and FTX or KLF13 was predicted by bioinformatics software (Starbase, http://starbase.sysu.edu.cn/; DIANA TOOL, http://snf-515788.vm.okeanos.grnet.gr/index.php?r=site/page&view=software) and determined by a dual-luciferase reporter assay. In brief, the wild-type FTX (FTX-WT), KLF13 (KLF13-WT), mutant-type FTX (FTX-MUT), and KLF13 (KLF13-MUT) luciferase vectors were constructed and separately co-transfected in H9C2 cells with miR-150 or miR-con. Followed by analysis with Dual-Luciferase^®^ Reporter Assay System, the relative luciferase activities were measured using a multimode reader (Tecan, Männedorf, Switzerland).

### 2.8 RNA immunoprecipitation (RIP) assay

RIP was conducted using the Magna RIP™ RNA-Binding Protein Immunoprecipitation kit (Millipore, MA, USA), according to the manufacturer’s instructions. H9C2 cells transfected with miR-150 or miR-con were lysed by RIP buffer. Next the cell lysate was incubated at 4°C overnight with magnetic beads coated with anti-Ago2 or IgG antibody (Millipore). Finally, the enrichment of FTX and KLF13 was detected by qRT-PCR.

### 2.9 Western blot

Western blot was conducted as previously reported [23]. In brief, total protein was extracted from H9C2 cells treated with 100 µM H_2_O_2_ and transfected with the vectors. The protein was then resolved by sodium dodecyl sulfate–polyacrylamide gel electrophoresis and transferred to polyvinylidene difluoride membranes (Millipore), which were then blocked with 5% nonfat milk. After that, the membranes were probed with primary antibodies against KLF13 (cat. no. ab190624; 1:1000; Abcam, Cambridge, UK) or β-actin (cat. no. ab8227; 1:3000; Abcam) and detected using HRP-conjugated secondary antibody (cat. no. D110058; 1:20000; Sangon, Shanghai, China). Protein bands were visualized using ECL detection reagent (Vazyme, Nanjing, China).

### 2.10 *In vivo* assay

Ten-week-old male C57BL/6 mice were purchased from Hubei Research Center of Laboratory Animal (Wuhan, China) and kept in an specific pathogen-free (SPF) environment. To evaluate the biological role of FTX, pcDNA vs FTX was delivered into the aortic root, as previously described [24]. At least five mice from each group survived *in vivo* and were used for subsequent analysis. Five days later, the I/R model was established by ligating the left anterior descending artery for 30 min. After 24 h of reperfusion, the heart was excised. And then, the area of infarction and a total area of the transverse section were measured using NIH Image J software, followed by analysis with qRT-PCR and Western blot.


**Ethical approval:** The research related to animal use has been complied with all the relevant national regulations and institutional policies for the care and use of animals and has been approved by the Institutional Animal Care and Use Committee of the First Affiliated Hospital of Airforce Military Medical University.

### 2.11 Statistical analysis

All the data were presented as mean ± standard deviation (SD). Statistical analysis was performed by GraphPad Prism 7 (San Diego, CA, USA). The correlation between miR-150 and FTX or KLF13 was analyzed by Pearson’s correlation coefficient. *P* values less than 0.05 (*P* < 0.05) were considered statistically significant.

## 3 Results

### 3.1 H_2_O_2_ inhibited cardiomyocyte cell proliferation and promoted cell apoptosis

**Figure 1:**
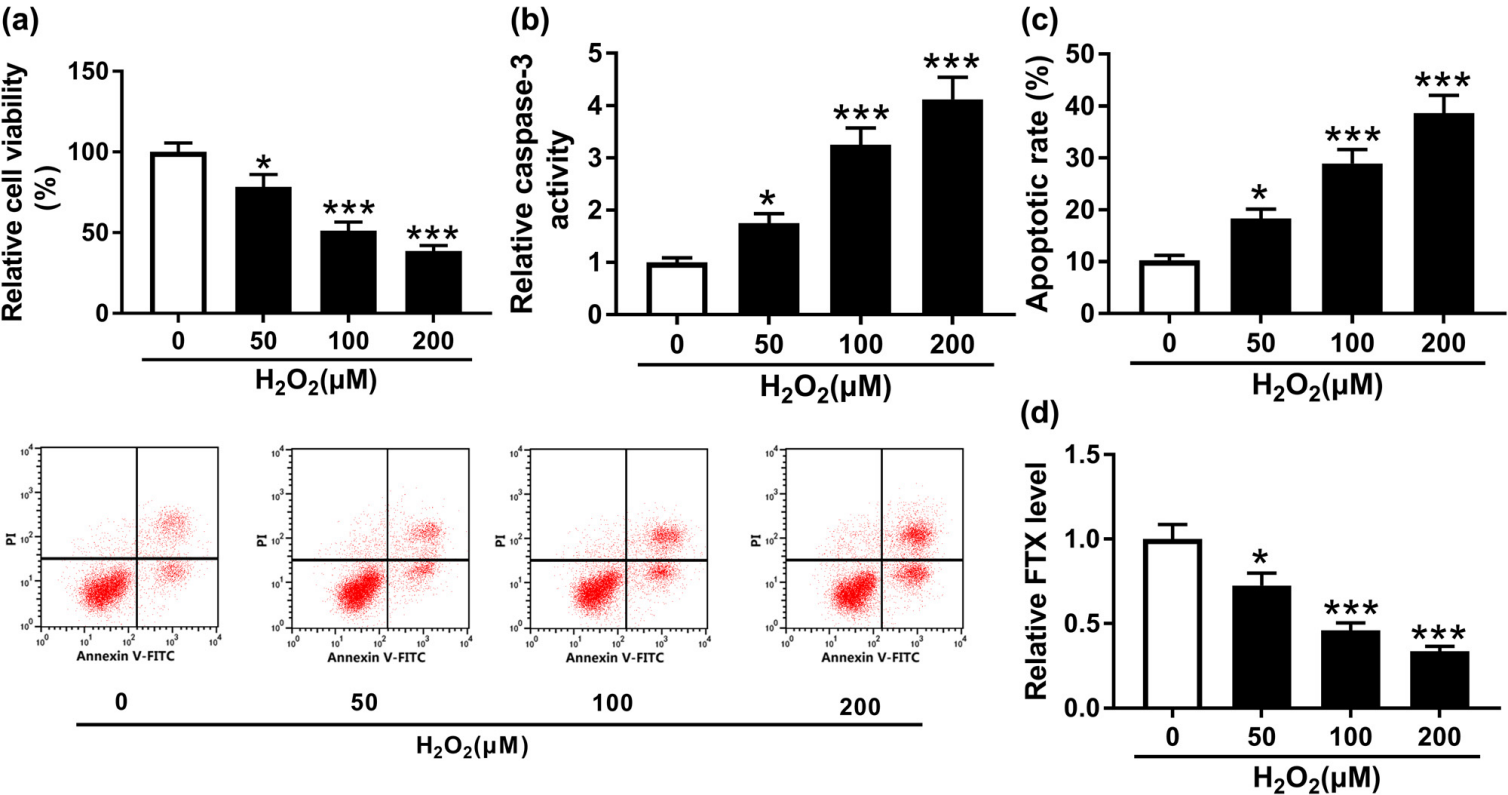
H_2_O_2_ repressed cell proliferation and facilitated cell apoptosis of cardiomyocytes. H9C2 cells were treated with H_2_O_2_ (0, 50, 100, and 200 µM) for 24 h. (a) Cell viability of H9C2 cells was measured by CCK-8 assay. (b) Caspase-3 activity of H9C2 cells was detected using caspase-3 activity detection kit. (c) Cell apoptosis of H9C2 cells was evaluated by flow cytometry. (d) FTX expression in H9C2 cells treated with H_2_O_2_ was assessed by qRT-PCR. *P < 0.05, ***P < 0.001, *n* = 3.

It is well acknowledged that H_2_O_2_ can induce cell death [25]. To explore the effects of H_2_O_2_ on cardiomyocyte growth and apoptosis, H9C2 cells were treated with H_2_O_2_ (0, 50, 100, and 200 µM) for 24 h. As illustrated in Figure 1a, H_2_O_2_ repressed cardiomyocyte proliferation in a dose-dependent manner. In addition, caspase-3 activity was enhanced by H_2_O_2_ treatment (Figure 1b). Meanwhile, we observed that H_2_O_2_ induced cardiomyocyte apoptosis in a dose-dependent manner (Figure 1c). To explore the underlying mechanism, FTX expression was evaluated by qRT-PCR. The result showed that FTX expression was reduced by H_2_O_2_ treatment (Figure 1d). Collectively, H_2_O_2_ may affect cardiomyocyte development by regulating FTX expression.

### 3.2 FTX abolished H_2_O_2_-induced suppression of proliferation and promotion of apoptosis of cardiomyocytes

**Figure 2:**
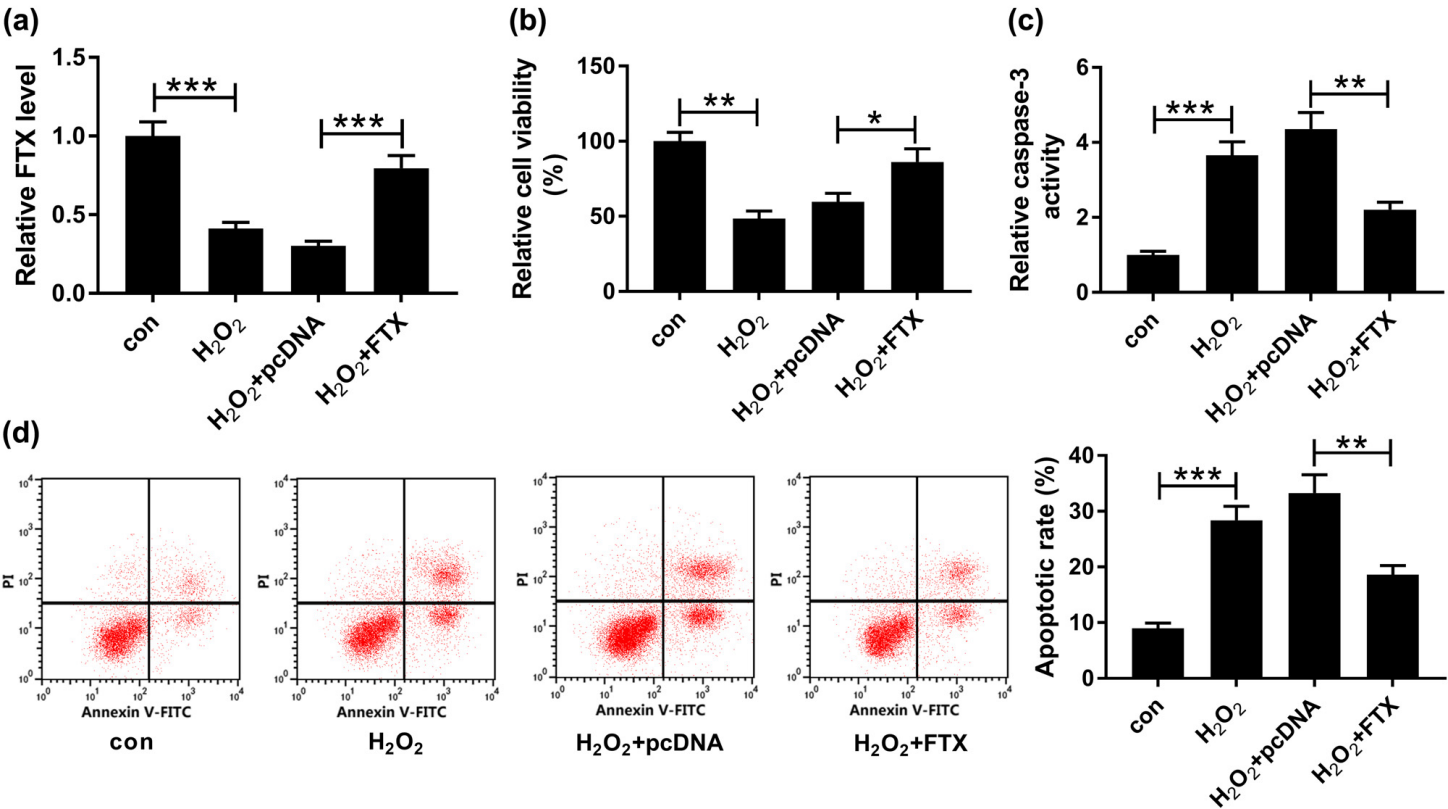
FTX attenuated H_2_O_2_-induced inhibition of cardiomyocyte cell proliferation and acceleration of apoptosis. H9C2 cells were treated with 100 µM H_2_O_2_ for 24 h and transfected with FTX for 48 h. (a) FTX expression was analyzed by qRT-PCR. (b) Detection of cell viability using CCK-8 assay. (c) Evaluation of caspase-3 activity by caspase-3 activity detection kit. (d) Analysis of cell apoptosis using flow cytometry. *P < 0.05, **P < 0.01, ***P < 0.001, *n* = 3.

**Figure A1:**
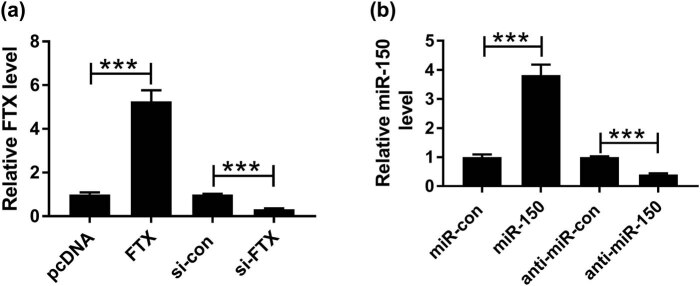
Transfection efficiency of pcDNA-FTX, si-FTX, miR-150, and anti-miR-150 in H9C2 cells. (a) FTX level was detected in H9C2 cells transfected with pcDNA, FTX, si-con, and si-FTX. (b) MiR-150 level was measured in H9C2 cells transfected with miR-con, miR-150, anti-miR-con, and anti-miR-150. ***P < 0.001, *n* = 3.

In order to investigate the effects of FTX on cell viability and apoptosis of H_2_O_2_-treated cardiomyocytes, untransfected or transfected H9C2 cells were treated with 100 µM H_2_O_2_ for 24 h. As displayed in Figure 2a, FTX expression was inhibited by H_2_O_2_, and the inhibition was reversed by transfection with FTX. The overexpression efficiency of FTX in H9C2 cells is shown in Figure A1. In addition, the overexpression of FTX restored H_2_O_2_-mediated inhibition of proliferation (Figure 2b) and promotion of apoptosis (Figure 2d) of H9C2 cells. In addition, the caspase-3 activity of H9C2 cells was examined by a caspase-3 activity detection kit. The result showed that the caspase-3 activity was enhanced by H_2_O_2_ and reduced by FTX (Figure 2c). Taken together, FTX produced regulatory effects opposite to those of H_2_O_2_ on cell proliferation and apoptosis of cardiomyocytes.

### 3.3 FTX directly targeted miR-150

**Figure 3:**
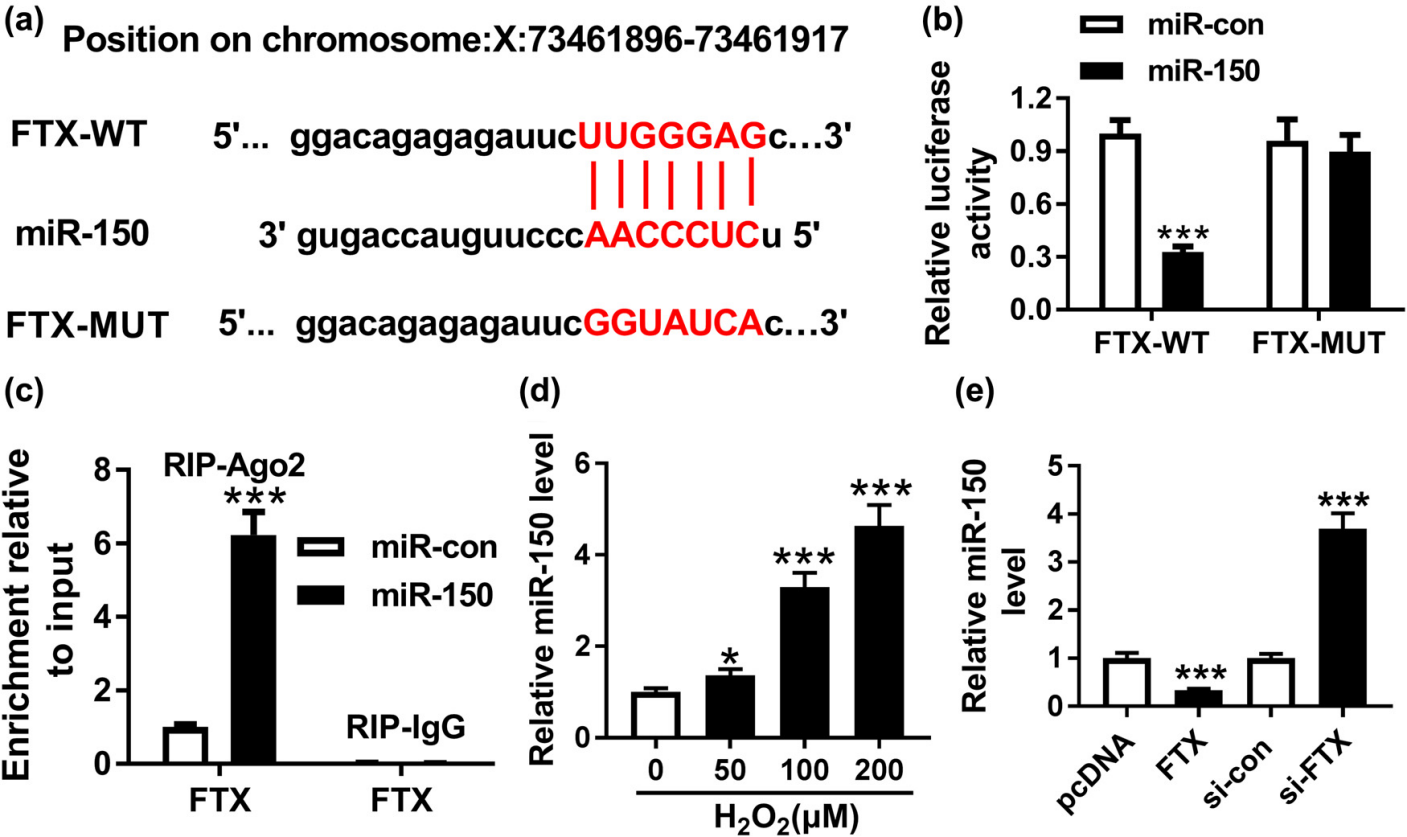
FTX directly interacted with miR-150. (a) Sequence complementarity between FTX and miR-150 was predicted by StarBase. (b) Luciferase activity of H9C2 cells co-transfected with FTX-WT or FTX-MUT and miR-150 or miR-con was determined by dual-luciferase reporter assay. (c) The enrichment of FTX in H9C2 cells transfected with miR-150 and miR-con was analyzed by RIP assay. (d) The expression of miR-150 in H9C2 cells treated with H_2_O_2_ was evaluated by qRT-PCR. (e) Determination of miR-150 expression in H9C2 cells transfected with pcDNA, FTX, si-con, and si-FTX using qRT-PCR. *P < 0.05, ***P < 0.001, *n* = 3.

As predicted by the bioinformatics analysis tool StarBase, we identified potential binding sites between FTX and miR-150 (Figure 3a). Reduced luciferase activity in H9C2 cells co-transfected with FTX-WT and miR-150 validated the interaction between FTX and miR-150 (Figure 3b). The transfection efficiency of miR-150 mimics in H9C2 cells is presented in Figure A1. Furthermore, the RIP assay result showed an increased enrichment of FTX in H9C2 cells transfected with miR-150 as compared to miR-con (Figure 3c). The influence of H_2_O_2_ on miR-150 expression was further investigated by qRT-PCR. H_2_O_2_ treatment increased miR-150 expression in a dose-dependent manner (Figure 3d). The knockdown efficiency of FTX in H9C2 cells is shown in Figure A1. Moreover, miR-150 expression was decreased by FTX and increased by FTX knockdown (Figure 3e). Therefore, we considered the possibility that miR-150 is a direct target of FTX.

### 3.4 FTX regulated H_2_O_2_-induced cytotoxicity of cardiomyocytes by targeting miR-150

**Figure 4:**
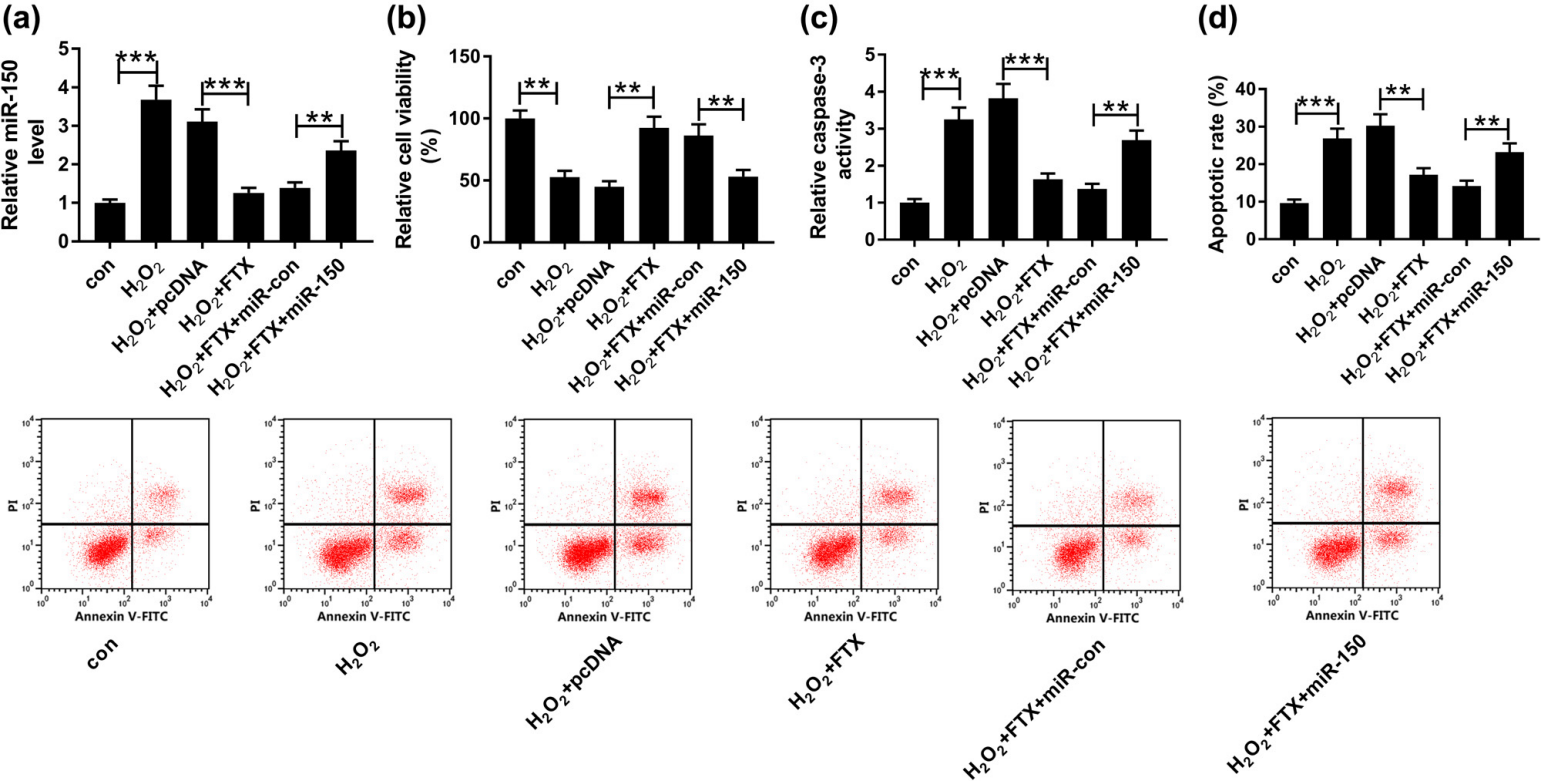
MiR-150 abrogated FTX-mediated promotion on proliferation and suppression on apoptosis of H_2_O_2_-treated cardiomyocytes. H9C2 cells were treated with 100 µM H_2_O_2_ for 24 h and transfected with pcDNA, FTX, FTX + miR-con and FTX + miR-150. (a) The expression of miR-150 was examined by qRT-PCR. (b) CCK-8 assay was employed to detect cell viability. (c) Analysis of caspase-3 activity using caspase-3 activity detection kit. (d) Examination of cell apoptosis by flow cytometry. **P < 0.01, ***P < 0.001, *n* = 3.

After demonstrating the interaction between FTX and miR-150, we predicted that FTX is able to regulate H_2_O_2_-induced cytotoxicity of cardiomyocytes by interacting with miR-150. To confirm this, H9C2 cells were transfected with pcDNA, FTX, FTX + miR-con, and FTX + miR-150 and then untransfected or transfected H9C2 cells were treated with 100 µM H_2_O_2_. As exhibited in Figure 4a, miR-150 transfection restored the repression of FTX on miR-150 expression in H_2_O_2_-treated H9C2 cells. More importantly, miR-150 neutralized the promotion of FTX on H_2_O_2_-induced cytotoxicity of H9C2 cells (Figure 4b). By contrast, both H_2_O_2_ and miR-150 were promoted, whereas FTX blocked caspase-3 activity in cardiomyocytes (Figure 4c). Consistently, miR-150 facilitated, whereas FTX attenuated cell apoptosis of cardiomyocytes with H_2_O_2_ treatment (Figure 4d). These findings revealed that FTX may modulate H_2_O_2_-induced cytotoxicity of cardiomyocytes by depleting miR-150.

### 3.5 KLF13 directly interacted with miR-150

**Figure 5:**
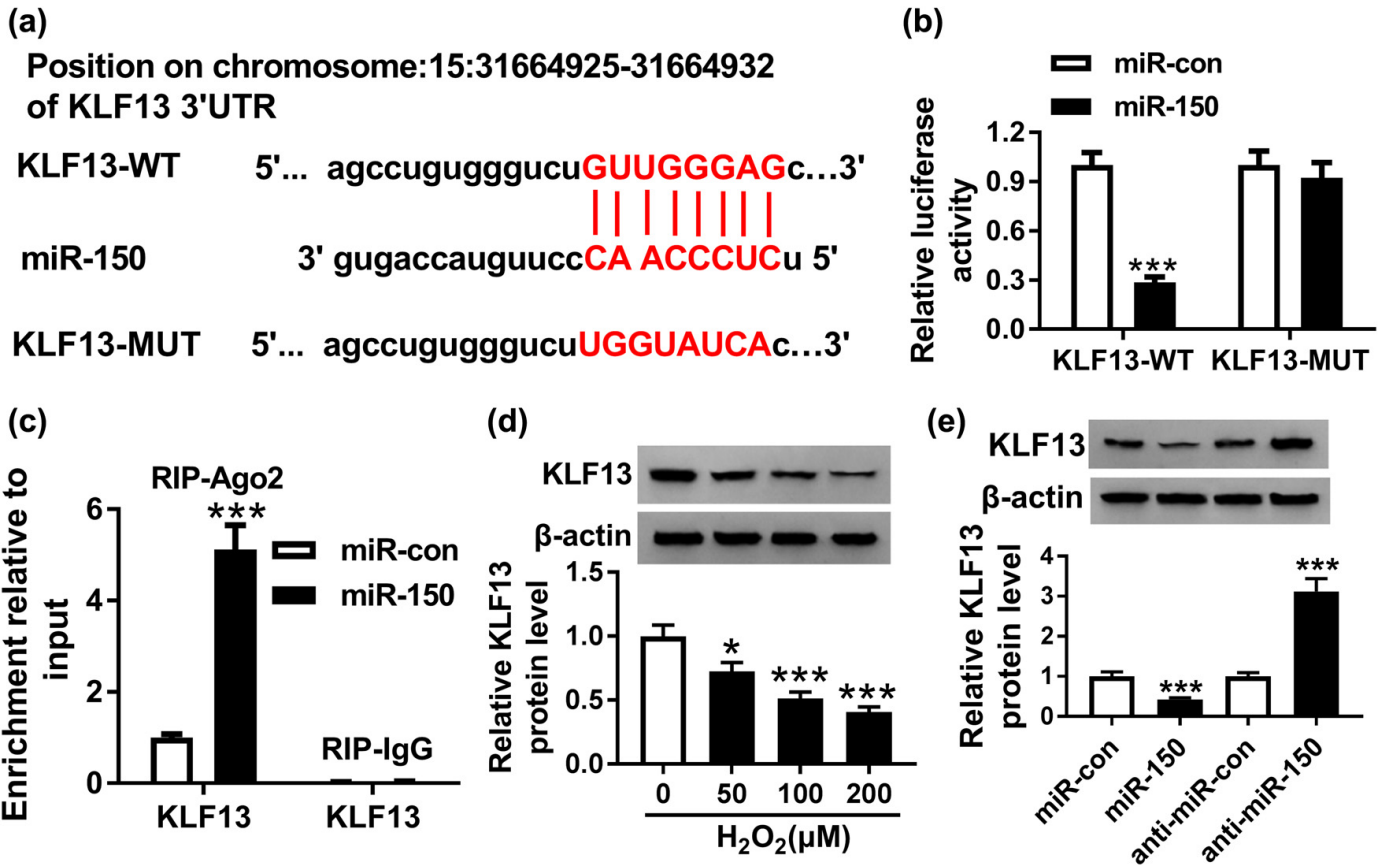
KLF13 was a target of miR-150. (a) Sequence complementarity between KLF13 and miR-150 was analyzed by DIANA TOOL. (b) Dual-luciferase reporter assay was used to measure luciferase activity of H9C2 cells co-transfected with KLF13-WT or KLF13-MUT and miR-150 or miR-con. (c) The enrichment of KLF13 in H9C2 cells transfected with miR-150 and miR-con was detected by RIP assay. (d) KLF13 protein expression in H9C2 cells treated with H_2_O_2_ was measured by Western blot. (e) KLF13 protein expression in H9C2 cells transfected with miR-con, miR-150, anti-miR-con, and anti-miR-150 was determined by Western blot. *P < 0.05, ***P < 0.001, *n* = 3.

Using DIANA TOOL, we discovered that miR-150 contained the binding sites of KLF13 (Figure 5a). To verify this prediction, the luciferase reporter system was constructed by co-transfecting KLF13-WT or KLF13-MUT and miR-150 or miR-con in H9C2 cells. Luciferase activity of H9C2 cells transfected with miR-150 was inhibited by KLF13-WT, thus confirming the interaction between miR-150 and KLF13 (Figure 5b). In addition, the enrichment of KLF13 was elevated by miR-150 in comparison to miR-con, as determined by the RIP assay (Figure 5c). As expected, KLF13 protein expression in H9C2 cells was repressed by H_2_O_2_ in a dose-dependent manner (Figure 5d). Transfection efficiency of miR-150 inhibitor in H9C2 cells is shown in Figure A1. Meanwhile, KLF13 protein expression was decreased by miR-150 and increased by miR-150 inhibitor (Figure 5e).

### 3.6 Elimination of KLF13 counteracted miR-150 inhibitor-mediated effects on proliferation and apoptosis in H_2_O_2_-treated cardiomyocytes

**Figure 6:**
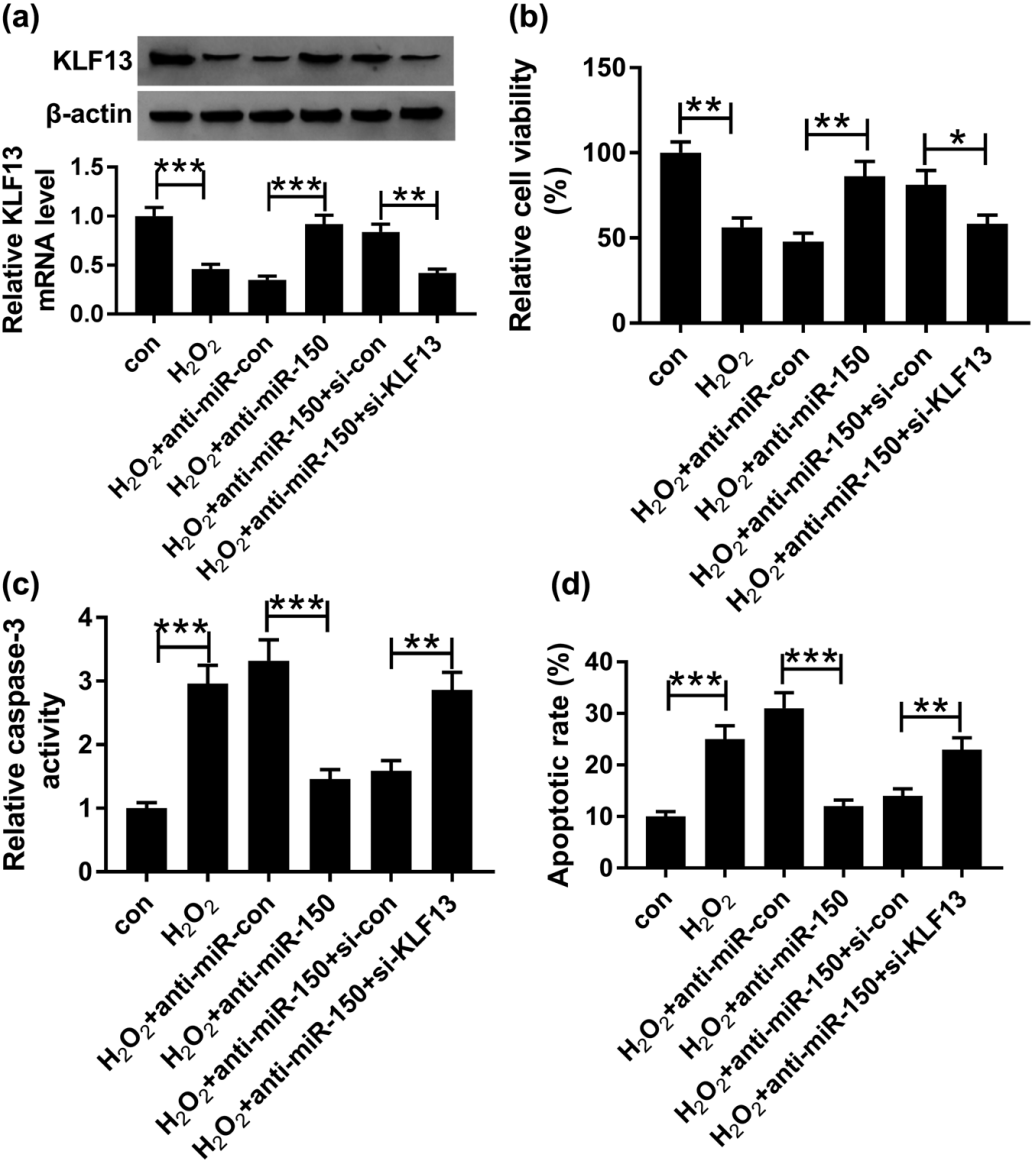
KLF13 silencing rescued miR-150 inhibitor-induced acceleration of proliferation and inhibition of apoptosis in cardiomyocytes treated with H_2_O_2_. H9C2 cells were treated with 100 µM H_2_O_2_ and transfected with anti-miR-con, anti-miR-150, anti-miR-150 + si-con, and anti-miR-150 + si-KLF13. (a) KLF13 mRNA and protein expression were measured by qRT-PCR and Western blot, respectively. (b) CCK-8 assay was used to measure cell viability. (c) Caspase-3 activity detection kit was applied to evaluate caspase-3 activity. (d) Cell apoptosis was determined by flow cytometry. *P < 0.05, **P < 0.01, ***P < 0.001, *n* = 3.

We further investigated the regulatory mechanism of the miR-150/KLF13 axis in H_2_O_2_-treated cardiomyocytes. H9C2 cells were transfected with anti-miR-con, anti-miR-150 (miR-150 inhibitor), anti-miR-150 + si-con, and anti-miR-150 + si-KLF13, followed by treatment with 100 µM H_2_O_2_. Quantitation of KLF13 expression at the mRNA and protein levels in H_2_O_2_-treated H9C2 cells revealed an increase mediated by miR-150 inhibitor and reduction by KLF13 knockdown (Figure 6a). Additionally, the depletion of KLF13 alleviated miR-150 inhibitor-induced enhancement of proliferation (Figure 6b) and suppression of apoptosis (Figure 6d) in H_2_O_2_-treated H9C2 cells. As expected, the caspase-3 activity of H_2_O_2_-treated H9C2 cells was enhanced by KLF13 silencing and reduced by miR-150 inhibitor (Figure 6c). In short, miR-150 may regulate H_2_O_2_-induced cytotoxicity of cardiomyocytes by targeting KLF13.

### 3.7 FTX regulated KLF13 expression by sponging miR-150 in cardiomyocytes

**Figure 7:**
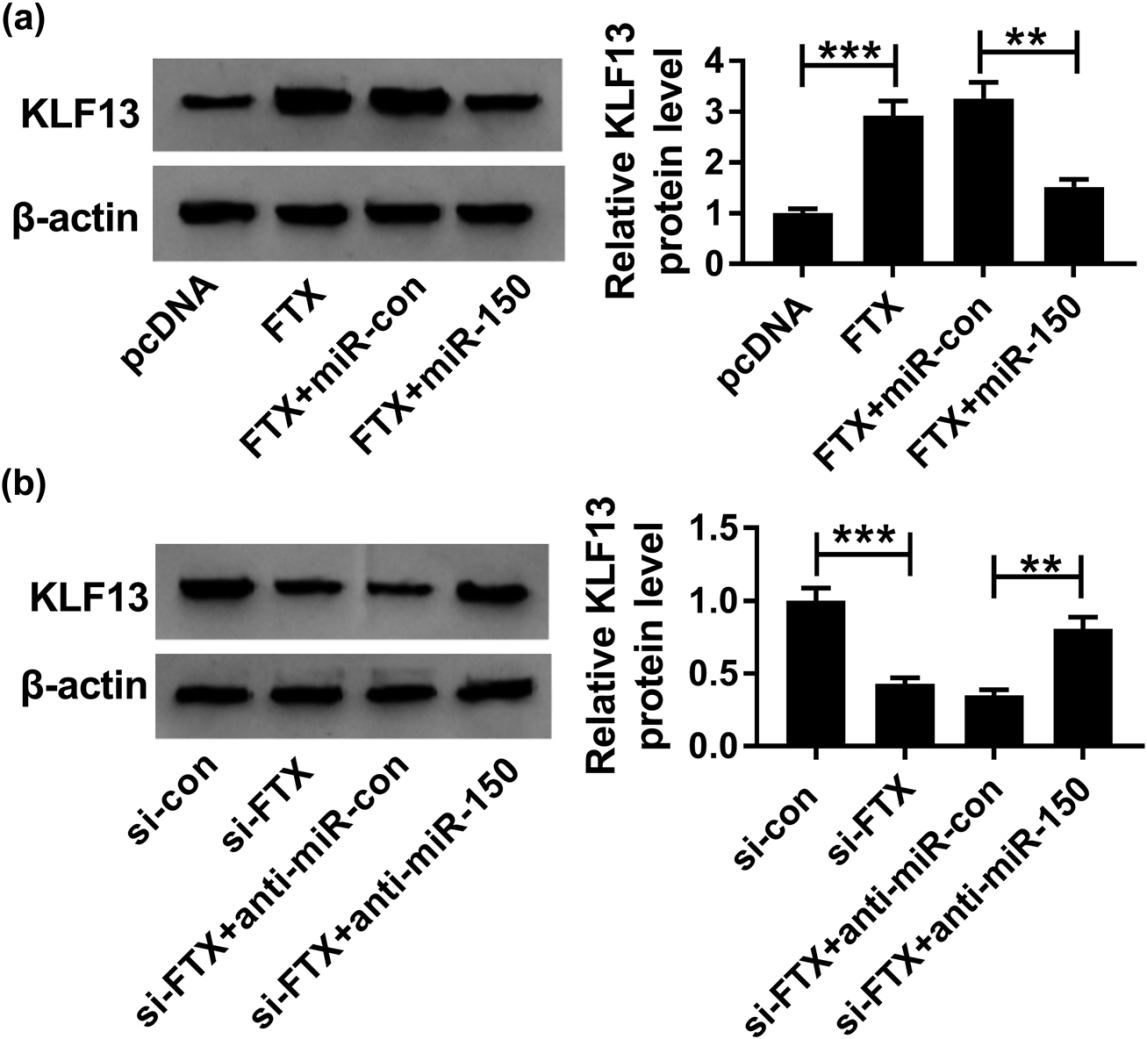
FTX increased the expression of KLF13 protein by depleting miR-150 in cardiomyocytes. (a) KLF13 protein expression in H9C2 cells transfected with pcDNA, FTX, FTX + miR-con and FTX + miR-150 was determined by Western blot. (b) KLF13 protein expression in H9C2 cells transfected with si-con, si-FTX, si-FTX + anti-miR-con, and si-FTX + anti-miR-150. **P < 0.01, ***P < 0.001, *n* = 3.

The specific molecular mechanism of FTX activity in cardiomyocytes was further explored by Western blot. As shown in Figure 7a, the expression of KLF13 protein was increased by FTX and inhibited by miR-150. Meanwhile, treatment with the miR-150 inhibitor reversed the inhibitory effect of FTX silencing on the KLF13 protein generation (Figure 7b). Therefore, FTX could sponge miR-150 and regulate KLF13 expression in cardiomyocytes.

### 3.8 FTX suppressed apoptosis in I/R-injured heart tissue *in vivo*

**Figure 8:**
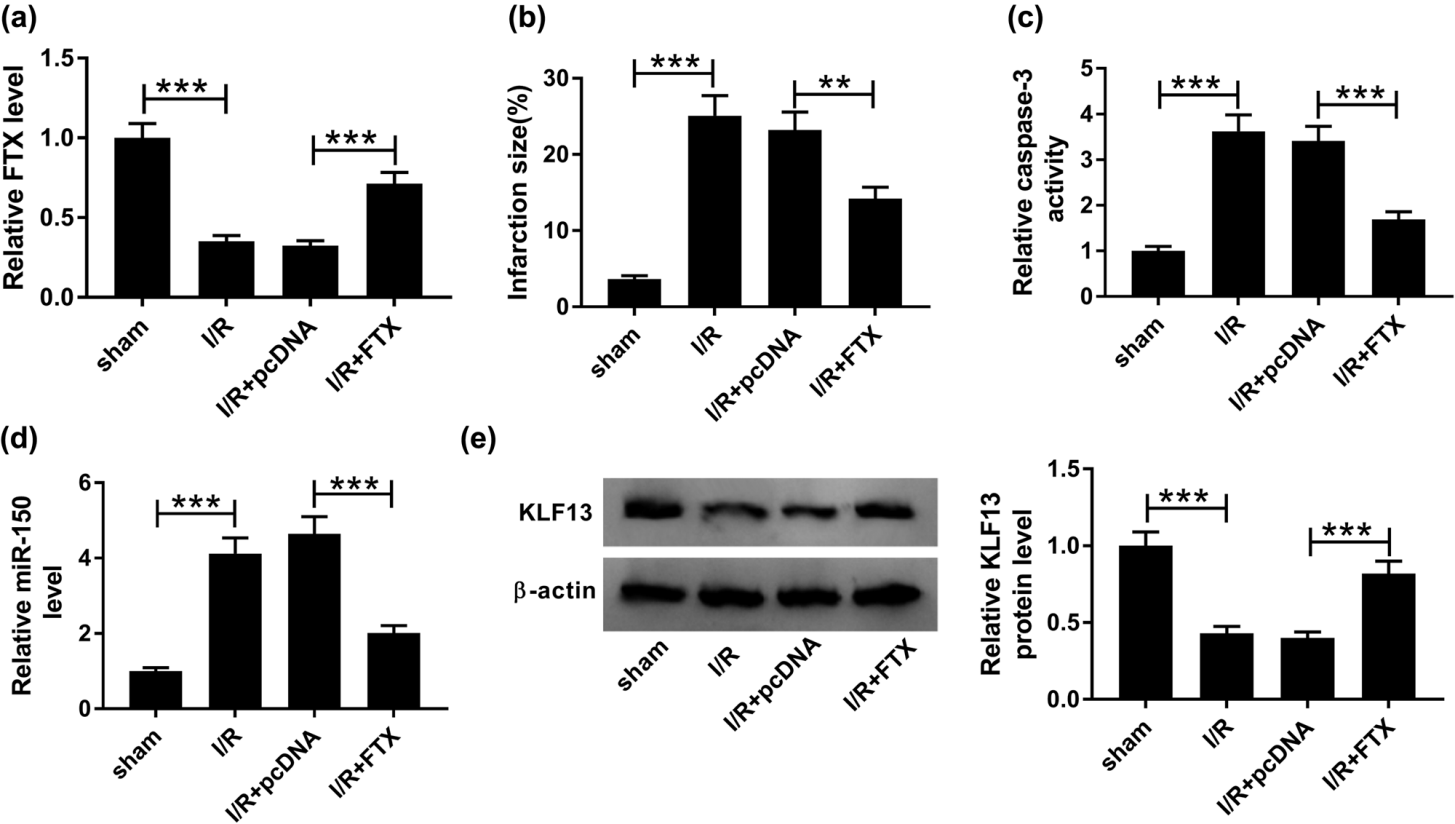
FTX overexpression inhibited infarction and apoptosis in I/R-injured heart tissue *in vivo*. (a) FTX level was detected in sham group and I/R groups (untransfected, transfected pcDNA, and transfected FTX) after reperfusion was measured by qRT-PCR. (b) Transections were measured for infarction size. (c) Caspase-3 activity was evaluated by caspase-3 activity detection kit. (d) MiR-150 level was detected by qRT-PCR assay. (e) KLF13 protein level was assessed by Western blot assay. **P < 0.01, ***P < 0.001, *n* = 3.

To assess the biological significance of FTX in hypoxia- or ischemia-challenged cardiomyocytes, we established an I/R model *in vivo*. As shown in Figure 8a, FTX expression was increased in the transfected-FTX I/R group compared with the transfected-pcDNA I/R group. Moreover, we found that the increased expression of FTX decreased the infarct size in I/R mouse *in vivo* (Figure 8b). We also detected the effect of pcDNA-FTX on caspase-3 activity. Our results suggest that FTX overexpression reduced caspase-3 activity in the I/R group *in vivo* relative to the transfected-pcDNA I/R group (Figure 8c). Also, miR-150 levels were decreased (Figure 8d), and KLF13 levels increased (Figure 8e) in the transfected-FTX I/R group compared with the transfected-pcDNA I/R group. These results suggest that FTX upregulation represses apoptosis in cardiomyocytes by regulating the miR-150/KLF13 axis in an *in vivo* model of I/R.

## 4 Discussion

Accumulating research has clarified that FTX is a significant predictor of different diseases, including cancers. Aberrant expression of FTX has been linked to the cell progression of various cancers. For instance, FTX indicated poor prognosis of colorectal cancer patients, and overexpression of FTX expedited the malignancy of colorectal cancer by accelerating cell growth [26]. The abundance of FTX is thought to influence cell viability, invasion, and aerobic glycolysis of hepatocellular carcinoma cells by regulating the PPARγ pathway [27]. Consistently, enhanced expression of FTX contributed to cell proliferation of hepatocellular carcinoma cells by interacting with miR-545 and regulating RIG-I expression [8]. However, FTX has also been shown to serve as a tumor suppressor by reducing cell progression in hepatocellular carcinoma by targeting miR-374a [28]. Moreover, FTX could regulate drug resistance of adriamycin against acute myeloid leukemia cells by competitively binding to miR-342 and altering ALG3 expression [29]. The regulatory effects of FTX in cardiomyocytes remain unclear.

Bioinformatics analysis prediction by StarBase indicated that FTX could potentially bind to miR-150. Reduced luciferase activity in H9C2 cells that were co-transfected with FTX-WT and miR-150 confirmed the interaction between FTX and miR-150. Previous studies demonstrated that miR-150 is a predictor of different diseases, such as neuropathic pain, heart failure, lung injury, and cancers [30,31,32]. For instance, miR-150 was reported to attenuate cell proliferation and accelerate cell apoptosis in Burkitt lymphoma by regulating LMO4 [33]. Similarly, miR-150 acted as a tumor suppressor in nasopharyngeal carcinoma to repress cell viability and G1/S phase transition by regulating CCND1 and CCNE2 [34]. In hepatocellular carcinoma, miR-150 is downregulated, and miR-150 weakened cell viability, colony formation, migration, and invasion by regulating the GAB1/ERK axis [35]. Therefore, we assumed that FTX participates in cell regulation of H_2_O_2_-treated cardiomyocyte by interacting with miR-150.

We initially validated the influence of H_2_O_2_ treatment on cardiomyocyte progression by CCK-8, flow cytometry, qRT-PCR, and caspase-3 activity detection assay. The results revealed that H_2_O_2_ weakened cell viability and stimulated cell apoptosis of cardiomyocytes, thus indicating that H_2_O_2_ is capable of inducing cardiomyocyte injury. Interestingly, we showed that FTX could protect cardiomyocytes from H_2_O_2_-induced cytotoxicity. The underlying molecular mechanism of FTX in cardiomyocyte protection was further investigated. The interaction between miR-150 and FTX or KLF13 was confirmed by a dual-luciferase reporter and RIP assay. Furthermore, H_2_O_2_ treatment enhanced the expression of miR-150 while reducing the expression of FTX or KLF13 in a dose-dependent manner. In addition, miR-150 abolished FTX-mediated improvement in proliferation and repression of apoptosis in H_2_O_2_-treated H9C2 cells. Likewise, KLF13 elimination relieved miR-150 inhibitor-mediated regulatory effects on cell progression in H_2_O_2_-treated cardiomyocytes. Also, we found that FTX could regulate KLF13 protein expression by absorbing miR-150 in cardiomyocytes. Additionally, FTX overexpression repressed apoptosis in I/R-stressed heart tissue *in vivo* by regulating the miR-150/KLF13 axis.

In summary, we revealed the molecular mechanism of FTX for cardiomyocyte protection after I/R injury. Our results demonstrated that FTX could alleviate H_2_O_2_-induced cardiomyocyte injury by increasing KLF13 expression through interacting with miR-150. This study demonstrated novel biomarkers for the diagnosis of AMI. One limitation of this study is that H9C2 cardiomyocytes may have different pathophysiological characteristics from human cardiomyocytes, thus suggesting further investigation. The emergence of cardiomyocytes derived from human-induced pluripotent stem cells and engineered cell culture platforms may provide more suitable tools for the establishment of cardiac I/R injury models.

## References

[1] Lu SF, Lu LX, Smith SC Jr, Dai X. Acute myocardial infarction in patients with paraplegia: characteristics, management, and outcomes. Am J Med. 2018;131:574 e1–11. 10.1016/j.amjmed.2017.11.045 29274759

[2] Pei W-N, Hu H-J, Liu F, Xiao B, Zuo Y-B, Cui W. C-reactive protein aggravates myocardial ischemia/reperfusion injury through activation of extracellular-signal-regulated kinase 1/2. J Geriatric Cardiol: JGC. 2018;15:492–503. 10.11909/j.issn.1671-5411.2018.07.001 PMC6198268 30364730

[3] Ritsinger V, Brismar K, Mellbin L, Nasman P, Ryden L, Soderberg S, et al. Elevated levels of insulin-like growth factor-binding protein 1 predict outcome after acute myocardial infarction: A long-term follow-up of the glucose tolerance in patients with acute myocardial infarction (GAMI) cohort. Diab Vasc Dis Res. 2018;15:387–95. 10.1177/1479164118781892 29992830

[4] Kumfu S, Charununtakorn ST, Jaiwongkam T, Chattipakorn N, Chattipakorn SC. Humanin exerts neuroprotection during cardiac ischemia-reperfusion injury. J Alzheimers Dis. 2018;61:1343–53. 10.3233/JAD-170708 29376862

[5] Kitazume-Taneike R, Taneike M, Omiya S, Misaka T, Nishida K, Yamaguchi O, et al. Ablation of Toll-like receptor 9 attenuates myocardial ischemia/reperfusion injury in mice. Biochem Biophys Res Commun. 2019;515:442–7. 10.1016/j.bbrc.2019.05.150 PMC6590932 31160091

[6] Qiao X, Jia S, Ye J, Fang X, Zhang C, Cao Y, et al. PTPIP51 regulates mouse cardiac ischemia/reperfusion through mediating the mitochondria-SR junction. Sci Rep. 2017;7:45379. 10.1038/srep45379 PMC5366942 28345618

[7] Tian YJ, Wang YH, Xiao AJ, Li PL, Guo J, Wang TJ, et al. Long noncoding RNA SBF2-AS1 act as a ceRNA to modulate cell proliferation via binding with miR-188-5p in acute myeloid leukemia. Artif Cell Nanomed Biotechnol. 2019;47:1730–7. 10.1080/21691401.2019.1608221 31062614

[8] Li X, Giri V, Cui Y, Yin M, Xian Z, Li J. LncRNA FTX inhibits hippocampal neuron apoptosis by regulating miR-21-5p/SOX7 axis in a rat model of temporal lobe epilepsy. Biochem Biophy Res Commun. 2019;512:79–86. 10.1016/j.bbrc.2019.03.019 30871773

[9] Cheng QY, Yang MC, Wu J, Jia XL, Xiao C, Lian T, et al. Reduced cardiac ischemia/reperfusion injury by hypothermic reperfusion via activation of transient receptor potential M8 channel. Life Sci. 2019;232:116658. 10.1016/j.lfs.2019.116658 31310758

[10] Liu X, Li C, Zhu J, Li W, Zhu Q. Dysregulation of FTX/miR-545 signaling pathway downregulates Tim-3 and is responsible for the abnormal activation of macrophage in cirrhosis. J Cell Biochem. 2019;120(2):2336–46. 10.1002/jcb.27562 30304545

[11] Zhang W, Bi Y, Li J, Peng F, Li H, Li C, et al. Long noncoding RNA FTX is upregulated in gliomas and promotes proliferation and invasion of glioma cells by negatively regulating miR-342-3p. Lab Invest. 2017;97:447–57. 10.1038/labinvest.2016.152 28112756

[12] He X, Sun F, Guo F, Wang K, Gao Y, Feng Y, et al. Knockdown of long noncoding RNA FTX inhibits proliferation, migration, and invasion in renal cell carcinoma cells. Oncol Res. 2017;25:157–66. 10.3727/096504016X14719078133203 PMC7840817 27983937

[13] Li N, Zhao X, Wang L, Zhang S, Cui M, He J. miR-494 suppresses tumor growth of epithelial ovarian carcinoma by targeting IGF1R. Tumour Biol. 2016;37:7767–76. 10.1007/s13277-015-4603-8 26695144

[14] Gao F, Wu H, Wang R, Guo Y, Zhang Z, Wang T, et al. MicroRNA-485-5p suppresses the proliferation, migration and invasion of small cell lung cancer cells by targeting flotillin-2. Bioengineered. 2019;10:1–12. 10.1080/21655979.2019.1586056 PMC6527069 30836864

[15] Sun H, Zhou X, Bao Y, Xiong G, Cui Y, Zhou H. Involvement of miR-4262 in paclitaxel resistance through the regulation of PTEN in non-small cell lung cancer. Open Biol. 2019;9:180227. 10.1098/rsob.180227 PMC6685930 31337279

[16] Neumann A, Napp LC, Kleeberger JA, Benecke N, Pfanne A, Haverich A, et al. MicroRNA 628-5p as a novel biomarker for cardiac allograft vasculopathy. Transplantation. 2017;101:e26–33. 10.1097/TP.0000000000001477 27653298

[17] Ma Y, Liu Y, Hou H, Yao Y, Meng H. MiR-150 predicts survival in patients with sepsis and inhibits LPS-induced inflammatory factors and apoptosis by targeting NF-kappaB1 in human umbilical vein endothelial cells. Biochem Biophys Res Commun. 2018;500:828–37. 10.1016/j.bbrc.2018.04.168 29689269

[18] Li P, Yao Y, Ma Y, Chen Y. MiR-150 attenuates LPS-induced acute lung injury via targeting AKT3. Int Immunopharmacol. 2019;75:105794. 10.1016/j.intimp.2019.105794 31398659

[19] Li H, Liu J, Cao W, Xiao X, Liang L, Liu-Smith F, et al. C-myc/miR-150/EPG5 axis mediated dysfunction of autophagy promotes development of non-small cell lung cancer. Theranostics. 2019;9:5134–48. 10.7150/thno.34887 PMC6691579 31410206

[20] Sun X, Zhang C, Cao Y, Liu E. miR-150 suppresses tumor growth in melanoma through downregulation of MYB. Oncol Res. 2019;27:317–23. 10.3727/096504018X15228863026239 PMC7848275 29690954

[21] Wu R, Yun Q, Zhang J, Bao J. Downregulation of KLF13 through DNMT1-mediated hypermethylation promotes glioma cell proliferation and invasion. OncoTargets Ther. 2019;12:1509–20. 10.2147/OTT.S188270 PMC6390852 30863117

[22] Bayoumi AS, Park K-M, Wang Y, Teoh J-P, Aonuma T, Tang Y, et al. A carvedilol-responsive microRNA, miR-125b-5p protects the heart from acute myocardial infarction by repressing pro-apoptotic bak1 and klf13 in cardiomyocytes. J Mol Cell Cardiol. 2018;114:72–82. 10.1016/j.yjmcc.2017.11.003 PMC5800989 29122578

[23] Song J, Wu X, Ma R, Miao L, Xiong L, Zhao W. Long noncoding RNA SNHG12 promotes cell proliferation and activates Wnt/beta-catenin signaling in prostate cancer through sponging microRNA-195. J Cell Biochem. 2019;120:13066–75. 10.1002/jcb.28578 30945357

[24] Liu C-Y, Zhang R-B, Li L-Y, Zhou T, An R-C, Zhang M, et al. LncRNA CAIF inhibits autophagy and attenuates myocardial infarction by blocking p53-mediated myocardin transcription. Nat Commun. 2018;9:29. 10.1038/s41467-017-02280-y PMC5750208 29295976

[25] Park WH. Exogenous H2O2 induces growth inhibition and cell death of human pulmonary artery smooth muscle cells via glutathione depletion. Mol Med Rep. 2016;14:936–42. 10.3892/mmr.2016.5307 27220315

[26] Guo X-B, Hua Z, Li C, Peng L-P, Wang J-S, Wang B, et al. Biological significance of long non-coding RNA FTX expression in human colorectal cancer. Int J Clin Exp Med. 2015;8(9):15591–600. PMC4658942 26629053

[27] Li X, Zhao Q, Qi J, Wang W, Zhang D, Li Z, et al. lncRNA Ftx promotes aerobic glycolysis and tumor progression through the PPARgamma pathway in hepatocellular carcinoma. Int J Oncol. 2018;53:551–66. 10.3892/ijo.2018.4418 PMC6017247 29845188

[28] Liu F, Yuan JH, Huang JF, Yang F, Wang TT, Ma JZ, et al. Long noncoding RNA FTX inhibits hepatocellular carcinoma proliferation and metastasis by binding MCM2 and miR-374a. Oncogene. 2016;35:5422–34. 10.1038/onc.2016.80 27065331

[29] Liu B, Ma X, Liu Q, Xiao Y, Pan S, Jia L. Aberrant mannosylation profile and FTX/miR-342/ALG3-axis contribute to development of drug resistance in acute myeloid leukemia. Cell Death Dis. 2018;9:688. 10.1038/s41419-018-0706-7 PMC5992136 29880818

[30] Lin X, Zhang S, Huo Z. Serum circulating miR-150 is a predictor of post-acute myocardial infarction heart failure. Int Heart J. 2019;60:280–6. 10.1536/ihj.18-306 30745540

[31] Cai W, Zhang Y, Liu Y, Liu H, Zhang Z, Su Z. Effects of miR-150 on neuropathic pain process via targeting AKT3. Biochem Biophys Res Commun. 2019;517:532–7. 10.1016/j.bbrc.2019.07.061 31376943

[32] Gan L, Sun T, Li B, Tian J, Zhang J, Chen X, et al. Serum miR-146a and miR-150 as potential new biomarkers for hip fracture-induced acute lung injury. Mediators Inflamm. 2018;2018:8101359. 10.1155/2018/8101359 PMC6230404 30510490

[33] Zhang D, Wei Y, Zhou J, Wang G, Xiao L, Xu J, et al. miR-150 might inhibit cell proliferation and promote cell apoptosis by targeting LMO4 in Burkitt lymphoma. J Cell Physiol. 2019;234:9652–62. 10.1002/jcp.27652 30422313

[34] Li X, Liu F, Lin B, Luo H, Liu M, Wu J, et al. miR-150 inhibits proliferation and tumorigenicity via retarding G1/S phase transition in nasopharyngeal carcinoma. Int J Oncol. 2017;50(4):1097–108. 10.3892/ijo.2017.3909 PMC5363880 28350089

[35] Sun W, Zhang Z, Wang J, Shang R, Zhou L, Wang X, et al. MicroRNA-150 suppresses cell proliferation and metastasis in hepatocellular carcinoma by inhibiting the GAB1-ERK axis. Oncotarget. 2016;7:11595–608. 10.18632/oncotarget.7292 PMC4905496 26871477

